# Inflammasome genetic variants are associated with tuberculosis, HIV-1 infection, and TB/HIV-immune reconstitution inflammatory syndrome outcomes

**DOI:** 10.3389/fcimb.2022.962059

**Published:** 2022-09-20

**Authors:** Nathalia Beatriz Ramos de Sá, Nara Cristina Silva de Souza, Milena Neira-Goulart, Marcelo Ribeiro-Alves, Tatiana Pereira Da Silva, Jose Henrique Pilotto, Valeria Cavalcanti Rolla, Carmem B. W. Giacoia-Gripp, Luzia Maria de Oliveira Pinto, Daniel Scott-Algara, Mariza Gonçalves Morgado, Sylvia Lopes Maia Teixeira

**Affiliations:** ^1^ 1Laboratory of AIDS & Molecular Immunology, Oswaldo Cruz Institute, FIOCRUZ, Rio de Janeiro, Brazil; ^2^ Laboratory of Clinical Research on STD/AIDS, National Institute of Infectious Diseases Evandro Chagas, FIOCRUZ, Rio de Janeiro, Brazil; ^3^ Nova Iguaçu General Hospital, Nova Iguaçu, Rio de Janeiro, Brazil; ^4^ Clinical Research Laboratory on Mycobacteria, National Institute of Infectious Diseases Evandro Chagas, FIOCRUZ, Rio de Janeiro, Brazil; ^5^ Laboratory of Viral Immunology, Oswaldo Cruz Institute, FIOCRUZ, Rio de Janeiro, Brazil; ^6^ Unité de Biologie Cellulaire des Lymphocytes, Institut Pasteur, Paris, France

**Keywords:** tuberculosis, HIV-1, TB-HIV/IRIS, inflammasome Single Nucleotide Polymorphism (SNP), proinflammatory cytokines

## Abstract

**Background:**

Tuberculosis (TB) and AIDS are the leading causes of infectious diseases death worldwide. Here, we investigated the relationship between from single nucleotide polymorphisms (SNPs) of the NLRP3, CARD8, AIM2, CASP-1, IFI16, and IL-1β inflammasome genes, as well as the profiles of secreted proinflammatory cytokines (e.g., IL-1β, IL-18, IL-33, and IL-6) with the TB clinical profiles, TB-HIV coinfection, and IRIS onset.

**Methods:**

The individuals were divided into four groups: TB-HIV group (n=88; 11 of them with IRIS), HIV-1 group (n=20), TB group (n=24) and healthy volunteers (HC) group (n=10), and were followed up at INI/FIOCRUZ and HGNI (Rio de Janeiro/Brazil) from 2006 to 2016. Real-time PCR was used to determine the genotypes of the Single Nucleotide Polymorphism (SNPs), and ELISA was used to measure the plasma cytokine levels. Unconditional logistic regression models were used to perform risk estimations.

**Results:**

A higher risk for extrapulmonary TB was associated with the TT genotype (aOR=6.76; P=0.026) in the NLRP3 rs4612666 Single Nucleotide Polymorphism (SNP) and the C-C-T-G-C haplotype (aOR=4.99; P= 0.017) in the NLRP3 variants. This same Single Nucleotide Polymorphism (SNP) was associated with lower risk against extrapulmonary TB when the carrier allele C (aOR=0.15; P=0.021) was present. Among those with HIV-1 infections, a higher risk for TB onset was associated with the GA genotype (aOR=5.5; P=0.044) in the IL1-β rs1143634 Single Nucleotide Polymorphism (SNP). In contrast, lower risk against TB onset was associated with the A-G haplotype (aOR=0.17; P= 0.026) in the CARD8 variants. Higher IL-6 and IL-33 levels were observed in individuals with TB. A higher risk for IRIS onset was associated with CD8 counts ≤ 500 cells/mm^3^ (aOR=12.32; P=0.010), the presence of extrapulmonary TB (aOR=6.6; P=0.038), and the CT genotype (aOR=61.06; P=0.026) or carrier allele T (aOR=61.06; P=0.026) in the AIM2 rs2276405 Single Nucleotide Polymorphism (SNP), whereas lower risk against IRIS onset was associated with the AT genotype (aOR=0.02; P=0.033) or carrier allele T (aOR=0.02; P=0.029) in the CARD8 rs2043211 Single Nucleotide Polymorphism (SNP) and the T-G haplotype (aOR=0.07; P= 0.033) in the CARD8 variants. No other significant associations were observed.

**Conclusions:**

Our results depict the involvement of genetic polymorphisms of crucial innate immunity genes and proinflammatory cytokines in the clinical outcomes related to TB-HIV coinfection.

## Introduction

Tuberculosis (TB) and AIDS are the major causes of death from infectious diseases worldwide. In 2020, 10 million TB cases were estimated globally, including 815,000 cases among people living with HIV (PLWH) (World Health Organization, 2020), making TB the most common comorbidity leading to death among PLWH (World Health Organization, 2020). In 2019, 16% of all cases of TB that were reported to the World Health Organization (WHO) were extrapulmonary TB (EPTB) (World Health Organization, 2020). Combined antiretroviral therapy (cART) during TB treatment improves survival by restoring immune functions (Müller et al., 2010). However, treatment with anti-TB drugs followed by cART initiation can lead to a paradoxical immune reconstitution inflammatory syndrome (IRIS) (Shelburne et al., 2005). Current research has established some pathological mechanisms that are related to IRIS development, such as high viral loads, low baseline CD4+ T-cell counts (<50–100 cells/mm3) (Antonelli et al., 2010; Luetkemeyer et al., 2014) with high levels of CD4 activation and replication (Tibúrcio et al., 2021), and short time intervals between TB treatment and cART (French et al., 2004; Chang et al., 2014; Tan et al., 2016). The genetic basis of host susceptibility to infectious diseases has received enormous attention (Fellay et al., 2009; Seaby et al., 2016; Wu et al., 2017). Highly polymorphic class I and II human leukocyte antigens (HLAs), killer-cell immunoglobulin-like receptor (KIR), cytokine genes, and genes involved in inflammation (inflammasome genes) are actively contributing factors that are associated with susceptibility and/or resistance to TB and HIV-1 infection (Kulkarni et al., 2008; Levy, 2009; Martin and Carrington, 2013; Pontillo et al., 2013; De Lima et al., 2016; Naranbhai and Carrington, 2017; Tsiara et al., 2018; de Sá et al., 2020). However, studies linking host genetics to the pathogenesis of IRIS are still scarce (Narendran et al., 2016; de Sá et al., 2020).

Inflammasomes are cytosolic multiprotein oligomers of the innate immune system that are responsible for the activation of inflammatory responses, including toll-like receptors (TLRs) and nod-like receptors (NLRs) that interact with several adaptor proteins, which leads to the activation of caspase-1 and induces the release of the proinflammatory cytokines such as IL-1ß and IL-18 (Rathinam and Fitzgerald, 2016). Different pattern-recognizing receptors (e.g., NLRP1 and NLRP3) can activate inflammasome assembly in response to specific stimuli, which leads to inflammation and the innate immune response (Man and Kanneganti, 2015). Dysregulation of the inflammasome has been associated with susceptibility to TB-HIV coinfection and TB-HIV/IRIS (Lai et al., 2015; Tan et al., 2015; Tan et al., 2016). In this regard, some investigations have found that TB-HIV/IRIS is associated with changes in the expression of cytokines that are related to the inflammasome activation pathway and other proinflammatory cytokines, such as IL-1β, IL-18, IL-33, IL-6, IL-17, IL-22, TNF, and IFN-γ, which suggests a key role in the development of TB-HIV/IRIS (Tadokera et al., 2011; Conesa-Botella et al., 2012; Tan et al., 2015; Tan et al., 2016; Ravimohan et al., 2018).

Although some studies have already observed the relationships among inflammasome coding genes and inflammasome activation-related cytokines with TB-HIV coinfected individuals, these studies are still scarce, especially for TB-HIV/IRIS individuals (Tan et al., 2016; Marais et al., 2017; Ravimohan et al., 2018; Ravimohan et al., 2020). In a previous study, we evaluated the role of host genetic markers (e.g., HLA-B, HLA-C, and KIR) in the risk and/or protection of TB-HIV coinfection outcomes, including the increased risk for TB-HIV/IRIS (de Sá et al., 2020). Considering the highly inflammatory profiles observed in TB-HIV coinfections, which increase during TB-HIV/IRIS, in the present study, we investigated the distributions of 11 single nucleotide polymorphisms (SNPs) of the major inflammasome pathway genes (e.g., NLRP3, CARD8, AIM2, CASP-1, IFI16, and IL-1β), cytokine levels (e.g., IL-1β, IL-6, IL-18, and IL-33), and their potential influence on the susceptibility to TB and/or HIV-1 as well as on the occurrence of TB-HIV/IRIS.

## Materials and methods

### Patient enrollment and study design

This study nested two clinical and immunological follow-up studies conducted in the Laboratory of AIDS & Molecular Immunology (IOC/FIOCRUZ) from 2006 to 2016, as previously described (da Silva et al., 2013; da Silva et al., 2017; Giacoia-Gripp et al., 2019). All participants signed an informed consent form, and the local ethics committee approved the studies. The study participants consisted of 142 individuals, who were divided into four groups as follows: individuals with TB and infected with HIV-1 (TB-HIV group, n=88; 11 of them with paradoxical TB-HIV/IRIS); individuals infected with HIV-1 without a diagnosis of TB (HIV-1 group, n=20); individuals with TB and seronegative for HIV-1 infection (TB group, n=24); and healthy controls with neither HIV-1 infection nor TB (HC, group, n=10).

The individuals were enrolled and followed up at the Clinical Research Laboratory on Mycobacteria (LAPCLINTB) of the National Institute of Infectious Diseases Evandro Chagas, Oswaldo Cruz Foundation (INI/FIOCRUZ), Rio de Janeiro, Brazil (2006-2011/2014-2016) and at the Nova Iguaçu General Hospital (HGNI), Rio de Janeiro, Brazil (2014-2016). The details regarding patient eligibility, enrollment, inclusion/exclusion criteria, anti-TB and cART treatments, study design, demographic and clinical data at the study entry visit, and availability of blood samples were previously described (de Sá et al., 2020). All TB-HIV coinfected individuals were investigated for the identification of IRIS development in both clinical centers. All IRIS cases observed in the study were classified as paradoxical, tuberculosis-associated IRIS, described as a worsening of TB signs and symptoms starting after cART initiation during TB treatment, mainly presenting enlargement of lymph nodes and inflammatory signs, not explained by any other diseases or by an adverse effect of drug therapy (Robertson et al., 2006; Meintjes et al., 2008), as previously detailed by our group (Demitto et al., 2019). In general, the IRIS cases included in the present study were self-resolving, or, if necessary, the patients were treated with corticoid-based therapy, such as Prednisone.

Skin color was self-declared following the classification system used by the Brazilian Institute of Geography and Statistics (IBGE) (Instituto Brasileiro de Geografia e Estatística, 2013) (which is an entity linked to the Brazilian Federal Government that is responsible for the official collection of statistical, geographic, cartographic, geodetic, and environmental information in Brazil).

### Genomic DNA extraction

DNA was extracted from whole blood using the QIAamp DNA Blood Mini Kit (Qiagen, Hilden, Nordrhein-Westfalen, Germany) according to the manufacturer’s instructions. The DNA concentrations were determined using a Thermo Scientific NanoDrop 2000 (Thermo Fisher Scientific, Waltham, Massachusetts, USA), and the filtrates containing the isolated DNA were stored at -20°C until genomic analyses.

### Single nucleotide polymorphism selection and genotyping

We selected 11 Single Nucleotide Polymorphism (SNPs) in six inflammasome genes by considering the relevance of each gene in the inflammasome pathway: CARD8 (rs2043211, rs6509365); AIM2 (rs2276405); IFI16 (rs1101996); CASP-1 (rs572687); IL-1β (rs1143634); and NLRP3 (rs10754558, rs1539019, rs4612666, rs3806268, and rs35829419). The Single Nucleotide Polymorphism (SNPs) were selected based on previous studies associating polymorphisms in inflammasome genes with HIV, tuberculosis, and HIV-TB (Pontillo et al., 2010; Pontillo et al., 2012; Pontillo et al., 2013). Single Nucleotide Polymorphism (SNP) genotyping was performed using commercially available TaqMan assays (Applied Biosystems/AB and Life Technologies) on the ABI7500 Real-Time platform (Applied Biosystems/AB and Life Technologies). Allelic discrimination was carried out employing Thermo Fisher Connect Software (Waltham, Massachusetts, EUA). The haplotype analyses were conducted by considering the most frequent haplotype of the NLRP3 (C-C-C-G-C haplotype) and CARD8 (AA) genes as the references. Detailed Single Nucleotide Polymorphism (SNP) information is provided in **Table S1**.

### Inflammatory cytokine plasma levels

The plasma concentrations of the proinflammatory cytokines included in the study were measured at study entry (baseline) before anti-TB and cART therapies, as follows: IL-1β/IL-1F2 (DuoSet ELISA Kit, R & D Systems, #DY201); IL-18 (Human Instant ELISA Kit, Thermo Fisher, BMS267INST); and IL-6 and IL-33 (Human Mini ABTS ELISA Development Kit, PeproTech, Inc., Rocky Hill, NJ) according to the manufacturer’s instructions. Standard curves were prepared by preparing serial dilutions of the aliquots that corresponded to the cytokine standards supplied by the manufacturers. Determination of the optical densities of samples and standards was performed using the BioTek ELx800TM absorbance microplate reader (BioTek® Instruments Inc., Vermont, USA) at wavelengths of either 405 or 450 nm, according to each protocol.

### Statistical analyses

For the descriptions of the patient samples included in the study, according to the sociodemographic, clinical, and laboratory characteristics among the individuals of the four groups, nonparametric Kruskal–Wallis rank-sum tests were used for continuous numerical variables, while Fisher’s exact tests were used for comparing the relative frequencies of the different levels of nominal/categorical variables. In the Single Nucleotide Polymorphism (SNP) analyses, the genotype frequencies were determined by direct count. The relative risks were described as adjusted odds ratios (aORs) with 95% CIs estimated through multiple unconditional logistic regression models. The log-transformed (base 10) least-squares mean differences of the plasma levels of cytokines that were measured by ELISA among the groups were estimated by fixed effects multiple linear regression models. The homozygous genotypes of the minor frequency allele (carriers) were compared with other genotypes (noncarriers) to observe better the differences caused by the variations. Adjustments to the confidence levels were made using Sidak’s method, and P-value adjustments for multiple comparisons were made using Tukey’s method whenever necessary. For both the cytokine serum levels and for the relative risk analysis, we included any clinical phenotypic marker that was associated with different outcomes as confounders in the modeling to eliminate any possible bias. All statistical analyses were performed using R version 4.1.3 (R Core Team, 2022).

## Results

### Sociodemographic, clinical, and laboratory characteristics

The sociodemographic, clinical, and laboratory characteristics of the 142 individuals included in the present study, which were categorized according to the presence or absence of TB, are listed in **Table 1**. Among the 88 TB-HIV coinfected individuals, 11 had paradoxical TB-HIV/IRIS. Most of the participants were males (73.9%). The overall proportions of individuals with white or brown skin color were equivalent (39.4% and 40.1%, respectively), which indicated that ethnicity was not dependent on group arrangement, which could influence the genetic analyses discussed here. Regarding educational levels, 43% of the individuals had lower secondary education, and 27.5% had an upper secondary education. No significant differences were observed among the groups (**Table 1**).

**Table 1:** Sociodemographic, clinical, and laboratory data for individuals included in the study categorized according to the presence or absence of TB.

Features	OverallN=142	All the groups		aOR^a^ (95%CI)	*P*-value^b^
		With TB N=112	Without TB N=30		
**Gender; n (%)**					
** Male**	105 (73.9%)	86 (76.79%)	19 (63.33%)	Reference	Reference
** Female**	37 (26.1%)	26 (23.21%)	11 (36.67%)	0.53 (0.22-1.3)	0.166
**SkinColor^c^; n (%)**					
** Brown**	57 (40.1%)	41 (36.61%)	16 (53.33%)	Reference	Reference
** Black**	29 (20.4%)	25 (22.32%)	4 (13.33%)	2.45 (0.71-8.43)	0.313
** White**	56 (39.4%)	46 (41.07%)	10 (33.33%)	1.88 (0.74-4.78)	0.313
**Education^d^; n (%)**					
** Bachelor**	9 (6.3%)	6 (5.36%)	3 (10%)	1.11 (0.22-5.67)	1
** Upper-secondary**	39 (27.5%)	27 (24.11%)	12 (40%)	0.72 (0.28-1.85)	1
** Lower-secondary**	61 (43%)	46 (41.07%)	15 (50%)	Reference	Reference
** Primary**	26 (18.3%)	26 (23.21%)	0 (0%)	NC	NC
** Unknown**	7 (4.9%)	7 (6.25%)	0 (0%)	NC	NC
**HIV status; n (%)**					
** Yes**	108 (76.1%)	88 (78.57%)	20 (66.67%)	Reference	Reference
** No**	34 (24.9%)	24 (21.43%)	10 (33.33%)	1.74 (0.19-15.67)	0.621
**CD4 count (cells/µL); n (%)**					
** ≤ 200 cells/µL**	87 (63%)	67 (62.04%)	20 (66.67%)	Reference	Reference
** > 200 cells/µL**	51 (37%)	41 (37.96%)	10 (33.33%)	11.37 (0.86-150.77)	0.065
**CD8 count (cells/µL); n (%)**					
** ≤ 500 cells/µL**	60 (45.1%)	44 (42.72%)	16 (53.33%)	Reference	Reference
** > 500 cells/µL**	73 (54.9%)	59 (57.28%)	14 (46.67%)	1.42 (0.61-3.3)	0.418
**CD4/CD8 ratio; n (%)**					
** ≤ 1**	106 (79.7%)	85 (82.52%)	21 (70%)	Reference	Reference
** > 1**	27 (20.3%)	18 (17.48%)	9 (30%)	0.31 (0.03-3.01)	0.311

^a^Odds ratios were adjusted by skin color, education, site of tuberculosis, HIV transmission route, and CD8 count. ^b^P-values were calculated using the unconditional logistic regression model. Associations were considered significant with a value of P < 0.05. ^c^Skin color categorization followed the classificatory system employed by the Brazilian Institute of Geography and Statistics (IBGE) (Instituto Brasileiro de Geografia e Estatística, 2013). ^d^Classification, according to the International Standard Classification of Education (ISCED) maintained by the United Nations Educational, Scientific and Cultural Organization (UNESCO). N, number of individuals in each group; TB, tuberculosis; %, Frequencies; aOR, adjusted odds ratio; 95% CI, 95% confidence interval. NC, not calculated.

We further analyzed the sociodemographic, clinical, and laboratory characteristics according to the clinical TB presentations of the individuals included in this study [pulmonary (PTB) *vs.* extrapulmonary TB (EPTB)] and according to the occurrence or absence of TB in PLWH. As depicted in **Tables S2** and **S3**, there were no statistically significant differences among the groups in either analysis.

The sociodemographic, clinical, and laboratory characteristics of TB-HIV coinfected individuals with and without IRIS are listed in **Table S4**. It is noteworthy that TB-HIV individuals with EPTB (ORadj=6.6; P=0.038) or CD8 ≤ 500 cells/mm3 (ORadj=12.32; P=0.010) values presented an increased risk for IRIS.

### Alleles, genotypes, and haplotypes of inflammasome genes

The genotype frequencies of the 11 Single Nucleotide Polymorphism (SNPs) analyzed in the present study were in Hardy-Weinberg equilibrium among the groups (**Table S1**). An unconditional logistic multiple regression model that compared the genotypes, alleles, carriers, or haplotype frequencies of the 11 Single Nucleotide Polymorphism (SNPs) between the TB and without TB groups did not show any statistical significance (data not shown).

Among PLWH with and without TB, the unconditional logistic multiple regression model that compared the genotypes, alleles, carriers, or haplotype frequencies of the 11 Single Nucleotide Polymorphism (SNPs) showed an increased risk for TB onset only for individuals with the G/A genotype (ORadj=5.5; P=0.044) in the IL-1β rs1143634 polymorphism (**Table 2** **)**. On the other hand, lower risk of TB onset among PLWH was associated with the CARD8 A-G haplotype (ORadj=0.17; P= 0.026) (**Table 2** **)**. Similar analyses were also conducted according to the different clinical TB presentations (PTB and EPTB) regardless of HIV-1 coinfection (**Table 2**), and an increased risk for EPTB was associated with carrying the T/T genotype (ORadj=6.76; P=0.026) in the NLPR3 rs4612666 polymorphism or the NLRP3 C-C-T-G-C haplotype (ORadj=4.99; P= 0.017). On the other hand, protection against EPTB was associated with carrier C (ORadj=0.15; P=0.021) in the NLPR3 rs4612666 polymorphism (**Table 2**). No significant associations were observed for other polymorphisms and outcomes.

**Table 2:** Unconditional logistic multiple regression model of risk and protection factors for TB and for distinct TB clinical presentations according to selected inflammasome SNP genetic profiles.

Gene SNP (rs)	Genotypes, alleles and haplotypes	PLWH					Site of TB				
		With TB N=88	Without TB N=24	aOR^a^	95%CI	P-value^b^	PTB N=63	EPTB N=49	aOR^a^	95%CI	P-value^b^
**IL-1β rs1143634**	G/G	62 (70.45%)	17 (85%)	Ref			43 (68.25%)	35 (71.43%)	Ref		
	A/A	2 (2.27%)	1 (5%)	1.08	0.05-24.34	0.961	1 (1.59%)	1 (2.04%)	1.3	0.08-22.29	0.857
	G/A	24 (27.27%)	2 (10%)	5.5	1.04-29.02	**0.044**	19 (30.16%)	13 (26.53%)	0.64	0.26-1.61	0.346
	G	148 (84.09%)	36 (90%)	Ref		0.094	105 (83.33%)	83 (84.69%)			
	A	28 (15.91%)	4 (10%)	1.14	0.98-1.33		21 (16.67%)	15 (15.31%)	0.94	0.78-1.12	0.490
	Non-Carrier-G	2 (2.27%)	1 (5%)	Ref			1 (1.59%)	1 (2.04%)			
	Carrier-G	86 (97.73%)	19 (95%)	1.28	0.07-23.73	0.870	62 (98.41%)	48 (97.96%)	0.68	0.04-11.57	0.791
	Non-Carrier-A	62 (70.45%)	17 (85%)	Ref			43 (68.25%)	35 (71.43%)			
	Carrier-A	26 (29.55%)	3 (15%)	4.22	0.97-18.42	0.055	20 (31.75%)	14 (28.57%)	0.68	0.28-1.65	0.388
**NLPR3** **rs4612666**	C/C	38 (45.24%)	6 (30%)	Ref			31 (51.67%)	19 (41.3%)	Ref		
	C/T	35 (41.67%)	12 (60%)	0.31	0.09-1.04	0.057	26 (43.33%)	18 (39.13%)	0.98	0.4-2.39	0.965
	T/T	11 (13.1%)	2 (10%)	0.63	0.1-3.94	0.618	3 (5%)	9 (19.57%)	6.76	1.26-36.23	**0.026**
	C	111 (66.07%)	24 (60%)	Ref			88 (73.33%)	56 (60.87%)	Ref		
	T	57 (33.93%)	16 (40%)	0.93	0.83-1.05	0.223	32 (26.67%)	36 (39.13%)	1.15	0.99-1.34	0.061
	Non-Carrier-C	11 (13.1%)	2 (10%)	Ref			3 (5%)	9 (19.57%)	Ref		
	Carrier-C	73 (86.9%)	18 (90%)	0.89	0.16-4.94	0.897	57 (95%)	37 (80.43%)	0.15	0.03-0.75	**0.021**
	Non-Carrier-T	38 (45.24%)	6 (30%)	Ref			31 (51.67%)	19 (41.3%)	Ref		
	Carrier-T	46 (54.76%)	14 (70%)	0.36	0.11-1.14	0.082	29 (48.33%)	27 (58.7%)	1.44	0.63-3.3	0.383
**NLRP3** **rs10754558** **rs1539019** **rs4612666** **rs3806268** **rs35829419**	CCTGC	25 (14.88%)	9 (22.5%)	0.58	0.16-2.19	0.423	6 (5.26%)	15 (17.05%)	4.99	1.33-18.71	**0.017**
**CARD8** **rs2043211** **rs6509365**	AG	4 (2.27%)	4 (10%)	0.17	0.03-0.81	**0.026**	4 (3.17%)	2 (2.04%)	0.52	0.09-3.05	0.467

^a^Odds ratios were adjusted by skin color, education, site of tuberculosis, HIV transmission route, and CD8 count. ^b^P-values were calculated using the unconditional logistic regression model. Associations were considered significant with a value of P < 0.05. N, number of individuals in each group; TB, tuberculosis; PLWH, people living with HIV; aOR, adjusted odds ratio; 95% CI, 95% confidence interval; Ref, Reference; PTB, Pulmonary TB; EPTB, Extrapulmonary TB, A, T, G, and C = each allele count, irrespective of the genotype. Carrier-A = total of genotypes with the A allele, Carrier-T = total of genotypes with T allele, Carrier-C = total of genotypes with the C allele, Carrier-G = total of genotypes with the G allele, Non-Carrier-A = total of genotypes without the A allele, Non-Carrier-T = total of genotypes without the T allele, Non-Carrier-C = total of genotypes without the C allele, Non-Carrier-G = total of genotypes without the G allele. Bold indicate statistically significant results.

### TB-HIV/IRIS and inflammasome-related markers

By comparing the TB-HIV coinfected individuals with and without IRIS in relation to the allelic frequencies of the 11 Single Nucleotide Polymorphism (SNPs) analyzed in the present study, an increased risk for IRIS was associated with the C/T genotype (ORadj=61.06; P=0.026) and carrier-T (ORadj=61.06; P=0.026) in the AIM2 rs2276405 polymorphism. Nevertheless, a trend of increased risk for IRIS was also associated with bearing the T allele (ORadj= 1.49; P= 0.050) in the same polymorphism. Otherwise, lower risk IRIS onset was associated with the A/T genotype (ORadj=0.02; P=0.033) or carrier-T (ORadj=0.02; P=0.029) in the CARD8 rs2043211 polymorphism and with the CARD8 T-G haplotype (ORadj=0.07; P= 0.033) (**Table 3**). No significant associations were observed for the other polymorphisms.

**Table 3:** Unconditional logistic multiple regression model of risk and protection factors for TB-HIV/IRIS among TB-HIV individuals.

GeneSNP (rs)	Genotypes, alleles and haplotypes	TB-HIV individuals		aOR^a^ (CI95%)	*P*-value^b^
		Without IRIS (N =77)	With IRIS (N = 11)		
**CARD8** **rs2043211**	A/A	43 (56.58%)	10 (90.91%)	Reference	
	A/T	25 (32.89%)	1 (9.09%)	0.02 (0-0.73)	**0.033**
	T/T	8 (10.53%)	0 (0%)	NC	NC
	A	111 (73.03%)	21 (95.45%)	Reference	
	T	41 (26.97%)	1 (4.55%)	0.9 (0.81-1)	0.060
	Non Carrier-A	8 (10.53%)	0 (0%)	Reference	
	Carrier-A	68 (89.47%)	11 (100%)	NC	NC
	Non Carrier-T	43 (56.58%)	10 (90.91%)	Reference	
	Carrier-T	33 (43.42%)	1 (9.09%)	0.02 (0-0.67)	**0.029**
**CARD8** **rs6509365**	A/A	40 (51.95%)	9 (81.82%)	Reference	
	A/G	29 (37.66%)	2 (18.18%)	0.13 (0.01-1.21)	0.073
	G/G	8 (10.39%)	0 (0%)	NC	NC
	A	109 (70.78%)	20 (90.91%)	Reference	
	G	45 (29.22%)	2 (9.09%)	0.93 (0.84-1.03)	0.175
	Non Carrier-A	8 (10.39%)	0 (0%)	Reference	
	Carrier-A	69 (89.61%)	11 (100%)	NC	NC
	Non Carrier-G	40 (51.95%)	9 (81.82%)	Reference	
	Carrier-G	37 (48.05%)	2 (18.18%)	0.12 (0.01-1.08)	0.058
**AIM2** **rs2276405**	C/C	72 (97.3%)	9 (90%)	Reference	
	C/T	2 (2.7%)	1 (10%)	61.06 (1.62-2294.92)	**0.026**
	C	146 (98.65%)	19 (95%)	Reference	
	T	2 (1.35%)	1 (5%)	1.49 (1-2.22)	**0.050**
	Non Carrier-C	74 (100%)	74 (100%)	Reference	
	Carrier-C	10 (100%)	10 (100%)	NC	NC
	Non Carrier-T	72 (97.3%)	9 (90%)	Reference	
	Carrier-T	2 (2.7%)	1 (10%)	61.06 (1.62-2294.92)	**0.026**
**CARD8** **rs2043211** **rs6509365**	TG	42 (27.27%)	1 (4.55%)	0.07 (0.01-0.81)	**0.033**

^a^Odds ratios were adjusted by skin color, education, site of tuberculosis, HIV transmission route, and CD8 count. ^b^P-values were calculated using the unconditional logistic regression model. Associations were considered significant with a value of P < 0.05. N, number of individuals in each group; TB, tuberculosis; aOR, adjusted odds ratio; 95% CI, 95% confidence interval. A, T, G, and C = each allele count, irrespective of the genotype. Carrier-A = total of genotypes with the A allele, Carrier-T = total of genotypes with T allele, Carrier-C = total of genotypes with the C allele, Carrier-G = total of genotypes with the G allele, Non-Carrier-A = total of genotypes without the A allele, Non-Carrier-T = total of genotypes without the T allele, Non-Carrier-C = total of genotypes without the C allele, Non-Carrier-G = total of genotypes without the G allele. NC, not calcuated. Bold indicate statistically significant results.

### Cytokines and inflammasome-related markers

**Figure 1:**
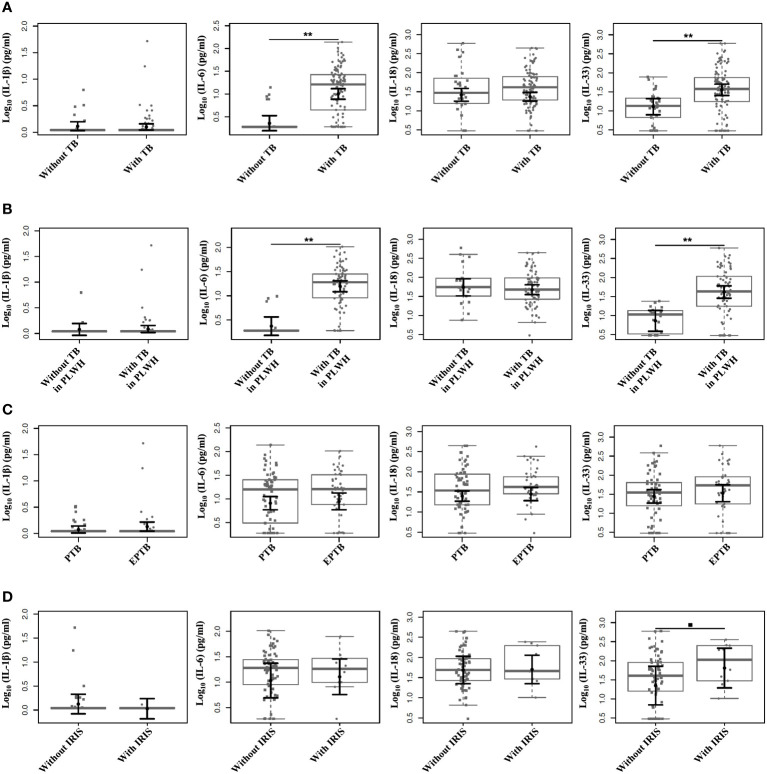
Comparison of plasma levels among the individuals included in the study categorized according to the outcomes. Plasma levels of IL-1β, IL-6, IL-18, and IL-33 measured by distinct ELISAs. **(A)** Comparison of plasma cytokine levels according to the presence or absence of TB. **(B)** Comparison of plasma cytokine levels according to the presence or absence of TB among PLWH. **(C)** Comparison of plasma cytokine levels according to different TB clinical presentations (PTB and EPTB). **(D)** Comparison of plasma cytokine levels according to the presence or absence of IRIS. Boxplots show the IQRs and sample medians (central solid gray line). Least-square means of log-transformed (base 10) levels of cytokines were pairwise compared among groups with T tests. Adjustments to the confidence levels were made by Sidak’s method, and P value adjustments were made by multiple comparisons using Tukey’s method. **P <0.0001; •P=0.073.

By comparing the plasma cytokine levels (IL-1β, IL-6, IL-18, and IL-33) among the groups and outcomes, we observed that the plasma levels of IL-6 and IL-33 were higher among individuals with TB than those without TB (P <0.0001 for both comparisons) (**Figure 1A**). Similarly, higher levels of IL-6 and IL-33 were observed in PLWH with TB than in those without TB (P <0.0001, for both comparisons) (**Figure 1B**). No differences in the IL-1β and IL-18 levels were observed among the analyzed groups. Similar analyses were also conducted according to the different clinical TB presentations (PTB and EPTB), and no statistically significant differences were observed among the groups (**Figure 1C**). Moreover, analyses of the IL-1β, IL-6, IL-18, and IL-33 plasma levels between individuals with and without IRIS showed that the mean IL-33 plasma levels were slightly higher among individuals with IRIS than among those without IRIS (P=0.073), indicating a trend for associating IL-33 plasma levels with TB-HIV/IRIS (**Figure 1D**). No statistical significance was observed for the plasma levels of the other cytokines (**Figure 1D**).

We next explored the relationships among the 11 Single Nucleotide Polymorphism (SNPs) and the plasma levels of the studied cytokines according to the carriers of the minor frequency allele (MFA) in the studied outcomes (with TB *vs*. without TB, PLWH with *vs*. without TB, PTB *vs*. EPTB, and with TB-HIV/IRIS *vs*. without IRIS) (**Table 4**). By comparing the carriers of the minor frequency allele in individuals with and without TB, CARD8 (rs2043211 and rs6509365), CASP-1 (rs572687), IFI16 (rs1101996), and NLRP3 (rs3806268, rs4612666, rs1539019, and rs10754558) had significant associations with the differences in IL-6 plasma levels; while IFI16 (rs1101996), and NLRP3 (rs3806268, rs1539019, and rs10754558) were significantly associated with differences in IL-33 plasma levels (**Table 4**). Among PLWH with and without TB, CARD8 (rs2043211 and rs6509365), CASP-1 (rs572687), IFI16 (rs1101996), IL-1β (rs1143634), and NLRP3 (rs3806268, rs4612666, rs1539019, and rs10754558) were significantly associated with differences in IL-6 plasma levels; while CARD8 (rs2043211 and rs6509365), IFI16 (rs1101996), and NLRP3 (rs3806268, rs4612666, rs1539019, and rs10754558) were significantly associated with differences in IL-33 plasma levels (**Table 4**). Among PTB and EPTB individuals, CARD8 (rs2043211 and rs6509365) was significantly associated with differences in IL-1β plasma levels (**Table 4**). Among individuals with and without IRIS, no statistically significant association was observed between the plasma cytokine levels and the minor frequency allele (**Table S5**).

**Table 4:** Plasma levels of cytokines according to the evaluated SNPs of the individuals included in the study.

Cytokines	Gene SNP (rs)	Carrier	Mean (CI95%)		P-value	Mean (CI95%)		P-value	Mean (CI95%)		P-value
			With TB	Without TB		With TB among HIV	Without TB among HIV		PTB	EPTB	
**IL-1β**	CARD8 rs2043211	A	0.142 (0.061 - 0.222)	0.202 (0.077 - 0.327)	0.842	0.151 (0.048 - 0.255)	0.129 (-0.063 - 0.321)	0.997	0.069 (-0.022 - 0.159)	0.319 (0.181 - 0.458)	**0.009**
	CARD8 rs6509365	A	0.139 (0.061 - 0.217)	0.165 (0.055 - 0.274)	0.981	0.141 (0.039 - 0.242)	0.102 (-0.046 - 0.251)	0.975	0.065 (-0.021 - 0.151)	0.301 (0.174 - 0.429)	**0.007**
	CASP-1 rs572687	G	0.055 (-0.039 - 0.149)	0.056 (-0.104 - 0.215)	1.000	0.039 (-0.082 - 0.159)	0.038 (-0.189 - 0.264)	1.000	0.025 (-0.114 - 0.163)	0.048 (-0.078 - 0.173)	0.994
	IFI16 rs1101996	C	0.083 (0.013 - 0.154)	0.081 (-0.032 - 0.194)	1.000	0.058 (-0.03 - 0.147)	-0.009 (-0.165 - 0.147)	0.864	0.06 (-0.025 - 0.145)	0.087 (-0.026 - 0.2)	0.977
	IL-1β rs1143634	G	0.1 (0.009 - 0.19)	0.042 (-0.125 - 0.208)	0.931	0.1 (-0.019 - 0.218)	0.058 (-0.235 - 0.352)	0.994	0.054 (-0.054 - 0.163)	0.176 (0.017 - 0.335)	0.573
	NLRP3 rs3806268	G	0.117 (0.044 - 0.191)	0.132 (0.003 - 0.26)	0.997	0.127 (0.039 - 0.214)	0.153 (-0.021 - 0.326)	0.993	0.052 (-0.039 - 0.143)	0.165 (0.059 - 0.271)	0.314
	NLRP3 rs35829419	C	0.048 (-0.135 - 0.232)	0.117 (-0.202 - 0.435)	0.982	0.06 (-0.238 - 0.357)	0.07 (-0.292 - 0.431)	1.000	0.032 (-0.201 - 0.264)	0.062 (-0.269 - 0.392)	0.999
	NLRP3 rs4612666	C	0.065 (-0.013 - 0.143)	0.091 (-0.016 - 0.198)	0.976	0.039 (-0.052 - 0.129)	0.047 (-0.091 - 0.186)	1.000	0.048 (-0.046 - 0.143)	0.053 (-0.064 - 0.17)	1.000
	NLRP3 rs1539019	C	0.092 (0.015 - 0.17)	0.068 (-0.062 - 0.197)	0.985	0.093 (0.001 - 0.185)	0.012 (-0.148 - 0.173)	0.798	0.073 (-0.029 - 0.174)	0.098 (-0.024 - 0.22)	0.987
	NLRP3 rs10754558	C	0.123 (0.054 - 0.193)	0.042 (-0.088 - 0.172)	0.652	0.109 (0.032 - 0.185)	0.039 (-0.118 - 0.197)	0.86	0.09 (-0.001 - 0.181)	0.158 (0.057 - 0.259)	0.684
**IL-6**	CARD8 rs2043211	A	0.953 (0.793 - 1.114)	0.353 (0.102 - 0.603)	**<0.001**	1.136 (0.966 - 1.307)	0.342 (0.024 - 0.659)	**<0.001**	0.84 (0.646 - 1.035)	1 (0.703 - 1.298)	0.775
	CARD8 rs6509365	A	0.899 (0.744 - 1.053)	0.385 (0.166 - 0.605)	**0.001**	1.071 (0.905 - 1.237)	0.45 (0.204 - 0.697)	**<0.001**	0.829 (0.641 - 1.018)	0.827 (0.555 - 1.1)	1.000
	CASP-1 rs572687	G	0.946 (0.756 - 1.135)	0.29 (-0.033 - 0.614)	**0.003**	1.15 (0.948 - 1.352)	0.289 (-0.094 - 0.671)	**0.001**	0.615 (0.329 - 0.901)	1.063 (0.803 - 1.322)	0.074
	IFI16 rs1101996	C	1.01 (0.869 - 1.15)	0.357 (0.133 - 0.581)	**<0.001**	1.185 (1.035 - 1.334)	0.354 (0.093 - 0.615)	**<0.001**	0.892 (0.715 - 1.069)	1.023 (0.788 - 1.258)	0.778
	IL-1β rs1143634	G	1.003 (0.827 - 1.178)	0.528 (0.197 - 0.858)	0.067	1.202 (1.012 - 1.392)	0.49 (-0.002 - 0.982)	**0.042**	0.978 (0.757 - 1.199)	0.867 (0.551 - 1.182)	0.934
	NLRP3 rs3806268	G	0.917 (0.774 - 1.061)	0.319 (0.073 - 0.565)	**<0.001**	1.095 (0.954 - 1.236)	0.391 (0.118 - 0.663)	**<0.001**	0.754 (0.571 - 0.938)	0.932 (0.72 - 1.145)	0.516
	NLRP3 rs35829419	C	0.726 (0.36 - 1.091)	0.155 (-0.478 - 0.789)	0.406	0.931 (0.434 - 1.429)	0.339 (-0.264 - 0.941)	0.432	0.578 (0.093 - 1.064)	0.857 (0.169 - 1.546)	0.916
	NLRP3 rs4612666	C	1.01 (0.855 - 1.166)	0.282 (0.07 - 0.494)	**<0.001**	1.226 (1.073 - 1.378)	0.386 (0.155 - 0.617)	**<0.001**	0.985 (0.789 - 1.182)	0.83 (0.591 - 1.069)	0.703
	NLRP3 rs1539019	C	0.913 (0.759 - 1.066)	0.317 (0.067 - 0.568)	**<0.001**	1.153 (1 - 1.307)	0.383 (0.12 - 0.646)	**<0.001**	0.838 (0.63 - 1.047)	0.86 (0.609 - 1.11)	0.999
	NLRP3 rs10754558	C	1.075 (0.939 - 1.211)	0.231 (-0.017 - 0.479)	**<0.001**	1.279 (1.156 - 1.402)	0.316 (0.072 - 0.56)	**<0.001**	1.03 (0.847 - 1.214)	0.987 (0.781 - 1.192)	0.985
**IL-18**	CARD8 rs2043211	A	1.363 (1.201 - 1.524)	1.333 (1.07 - 1.595)	0.997	1.591 (1.395 - 1.788)	1.684 (1.293 - 2.076)	0.975	1.329 (1.149 - 1.509)	1.588 (1.313 - 1.864)	0.345
	CARD8 rs6509365	A	1.31 (1.155 - 1.465)	1.417 (1.191 - 1.643)	0.857	1.516 (1.33 - 1.703)	1.773 (1.483 - 2.062)	0.467	1.28 (1.109 - 1.45)	1.523 (1.277 - 1.77)	0.303
	CASP-1 rs572687	G	1.333 (1.146 - 1.519)	1.43 (1.112 - 1.748)	0.947	1.69 (1.463 - 1.917)	1.667 (1.241 - 2.094)	1.000	1.303 (1.036 - 1.571)	1.458 (1.215 - 1.701)	0.801
	IFI16 rs1101996	C	1.361 (1.224 - 1.498)	1.413 (1.185 - 1.641)	0.976	1.695 (1.53 - 1.86)	1.636 (1.332 - 1.941)	0.986	1.418 (1.259 - 1.577)	1.381 (1.17 - 1.593)	0.991
	IL-1β rs1143634	G	1.548 (1.379 - 1.718)	1.354 (1.034 - 1.673)	0.716	1.826 (1.615 - 2.036)	1.668 (1.125 - 2.211)	0.95	1.617 (1.423 - 1.811)	1.522 (1.244 - 1.799)	0.939
	NLRP3 rs3806268	G	1.353 (1.21 - 1.496)	1.336 (1.072 - 1.6)	0.999	1.684 (1.52 - 1.847)	1.72 (1.365 - 2.076)	0.998	1.36 (1.188 - 1.533)	1.466 (1.266 - 1.665)	0.82
	NLRP3 rs35829419	C	1.301 (0.939 - 1.664)	1.37 (0.742 - 1.999)	0.998	1.338 (0.787 - 1.889)	1.77 (1.1 - 2.439)	0.751	1.497 (1.059 - 1.936)	1.014 (0.391 - 1.636)	0.6
	NLRP3 rs4612666	C	1.424 (1.273 - 1.576)	1.458 (1.243 - 1.674)	0.993	1.734 (1.565 - 1.903)	1.768 (1.493 - 2.042)	0.997	1.468 (1.289 - 1.648)	1.44 (1.222 - 1.657)	0.996
	NLRP3 rs1539019	C	1.345 (1.193 - 1.498)	1.39 (1.126 - 1.654)	0.989	1.611 (1.441 - 1.782)	1.725 (1.415 - 2.035)	0.91	1.345 (1.155 - 1.536)	1.459 (1.23 - 1.688)	0.846
	NLRP3 rs10754558	C	1.43 (1.294 - 1.565)	1.606 (1.341 - 1.871)	0.602	1.718 (1.577 - 1.859)	1.949 (1.642 - 2.257)	0.531	1.478 (1.308 - 1.648)	1.471 (1.281 - 1.662)	1.000
**IL-33**	CARD8 rs2043211	A	1.346 (1.142 - 1.55)	1.076 (0.757 - 1.394)	0.476	1.419 (1.172 - 1.665)	0.706 (0.248 - 1.164)	**0.038**	1.13 (0.896 - 1.365)	1.605 (1.246 - 1.964)	0.094
	CARD8 rs6509365	A	1.38 (1.184 - 1.577)	1.024 (0.745 - 1.303)	0.146	1.447 (1.208 - 1.685)	0.796 (0.441 - 1.151)	**0.018**	1.167 (0.941 - 1.393)	1.592 (1.265 - 1.919)	0.105
	CASP-1 rs572687	G	1.616 (1.372 - 1.861)	1.194 (0.777 - 1.61)	0.275	1.635 (1.346 - 1.925)	0.89 (0.344 - 1.437)	0.07	1.431 (1.057 - 1.805)	1.606 (1.267 - 1.946)	0.883
	IFI16 rs1101996	C	1.585 (1.406 - 1.764)	1.075 (0.789 - 1.361)	**0.008**	1.628 (1.415 - 1.84)	0.806 (0.436 - 1.176)	**0.001**	1.517 (1.294 - 1.74)	1.513 (1.217 - 1.808)	1.000
	IL-1β rs1143634	G	1.501 (1.274 - 1.728)	1.266 (0.838 - 1.694)	0.778	1.574 (1.302 - 1.847)	0.85 (0.143 - 1.556)	0.234	1.36 (1.08 - 1.641)	1.6 (1.199 - 2.001)	0.748
	NLRP3 rs3806268	G	1.544 (1.357 - 1.731)	1.035 (0.714 - 1.356)	**0.03**	1.608 (1.399 - 1.816)	0.863 (0.46 - 1.265)	**0.008**	1.35 (1.111 - 1.588)	1.583 (1.306 - 1.859)	0.51
	NLRP3 rs35829419	C	1.472 (0.995 - 1.95)	1.257 (0.429 - 2.085)	0.969	1.56 (0.844 - 2.276)	1.274 (0.406 - 2.141)	0.957	1.41 (0.786 - 2.034)	1.517 (0.632 - 2.403)	0.997
	NLRP3 rs4612666	C	1.514 (1.313 - 1.715)	1.097 (0.824 - 1.371)	0.051	1.576 (1.361 - 1.792)	0.969 (0.642 - 1.295)	**0.013**	1.393 (1.14 - 1.647)	1.475 (1.167 - 1.783)	0.971
	NLRP3 rs1539019	C	1.572 (1.372 - 1.772)	1.057 (0.731 - 1.383)	**0.022**	1.673 (1.454 - 1.892)	0.893 (0.517 - 1.268)	**0.002**	1.457 (1.19 - 1.724)	1.555 (1.235 - 1.875)	0.958
	NLRP3 rs10754558	C	1.662 (1.488 - 1.837)	0.96 (0.641 - 1.279)	**<0.001**	1.709 (1.528 - 1.889)	0.858 (0.5 - 1.216)	**<0.001**	1.602 (1.37 - 1.835)	1.572 (1.312 - 1.832)	0.997

P-values were calculated using the unconditional logistic regression model. Associations were considered significant with a value of P < 0.05. TB, tuberculosis; aOR, adjusted odds ratio; 95% CI, 95% confidence interval; PTB, Pulmonary TB; EPTB, Extrapulmonary TB. The AIM2 rs2276405 polymorphisms had insufficient observations and/or no way to calculate the standard error (all observations from one or more groups were equal to the lower detection limit of the assay) for the analyses between the groups with vs. without TB, with TB vs. without TB among HIV and PTB vs. EPTB. A, A allele; C, C allele; G, G allele. Bold indicate statistically significant results.

## Discussion

Innate immunity and inflammation are biological mechanisms with important roles in susceptibility to or protection from HIV infection and/or TB-related outcomes (Salie et al., 2015; Ravimohan et al., 2018; Zhao et al., 2019). Aberrantly high inflammasome activation and its signaling in different cells and tissues lead to several inflammatory pathologies, including IRIS (Chang et al., 2014; Marais et al., 2017). TB-associated IRIS (TB-IRIS) incidence ranges from 4% to 54% in different populations (Bana et al., 2016). In the studies conducted by our group, the incidence of TB-HIV/IRIS was determined to be approximately 12% (Serra et al., 2007). The low incidence of TB-HIV/IRIS may be due to the introduction of antiretroviral therapy for newly diagnosed TB-HIV individuals in Brazil, who still had higher CD4 levels when at the time when they were recruited and included in this study. Beyond the very low CD4 counts (<100/mm3) and short time intervals between the initiation of anti-TB and antiretroviral therapies (Laureillard et al., 2013), the discrepancies in the IRIS frequencies could also be attributed to difficulties in clinical diagnosis (no specificity of symptoms) or differences in the genetic backgrounds among the populations included in the studies (Bourgarit et al., 2006; Wilkinson et al., 2015). Indeed, a previous study conducted by our group showed that the HLA-B*41 allele, KIR2DS2, and the combination of KIR/HLA-C pairs were associated with an increased risk of TB-HIV/IRIS onset (de Sá et al., 2020).

Here, we showed that the C/T genotype (ORadj=61.06; P=0.026) or carrier-T (ORadj=61.06; P=0.026) in the AIM2 rs2276405 polymorphism was associated with an increased risk of TB-HIV/IRIS in TB-HIV individuals, whereas lower risk IRIS onset was associated with the A/T genotype (ORadj=0.02; P=0.033) or carrier-T (ORadj=0.02; P=0.029) in the CARD8 rs2043211 polymorphism and with the CARD8 T-G haplotype (ORadj=0.07; P= 0.033).

Absent in melanoma 2 (AIM2) is a cytosolic sensor for double-stranded DNA (dsDNA) and tumor suppressor that is responsible for inflammasome activation and is involved in the host immune response to viruses and intracellular bacteria (Saiga et al., 2012). AIM2 binds to HIV dsDNA and may trigger acute inflammation and pyroptosis (Ekabe et al., 2021). Regarding the AIM2 rs2276405 polymorphism, to our knowledge, only one study showed a significant difference in the genotype frequencies of this Single Nucleotide Polymorphism (SNP) between individuals with and without TB in a Taiwanese population (Liu et al., 2020). In our study, no association between AIM2 polymorphisms and the occurrence of TB or its clinical presentations or inflammasome-related cytokines was observed.

CARD8 negatively regulates the expression of the NLRP3 inflammasome by inhibiting the oligomerization of this receptor in unstimulated cells (Tangi et al., 2012; Ito et al., 2014). The CARD8 gene rs2043211 polymorphism is an A to T transversion on the template strand (Ko et al., 2009). A Brazilian study found an association between the CARD8 rs6509365 polymorphism and susceptibility to TB-HIV coinfection (Pontillo et al., 2013). This effect was stronger when this Single Nucleotide Polymorphism (SNP) was combined with the CARD8 rs2043211 polymorphism, supporting a novel association between the CARD8 gene and TB-HIV coinfection (Pontillo et al., 2013). However, in our study we did not observe any association of both CARD8 polymorphisms with TBHIV coinfection. However, when analyzing the CARD8 haplotypes a lower risk of TB onset among PLWH was observed. Moreover, a lower risk of IRIS associated with the CARD8 rs2043211 polymorphism and CARD8 haplotype was detected in our study. We also observed that carrying the MAF of both CARD8 polymorphisms was associated with increased levels of IL-6 in TB individuals compared to those without TB, IL-1β for those with EPTB clinical presentations, and IL-33 for TB-HIV cases.

Concerning the NLRP3 polymorphisms analyzed here, an increased risk for EPTB was associated with the TT genotype in the NLPR3 rs4612666 polymorphism or the C-C-T-G-C NLRP3 haplotype, whereas carrier-C in the NLRP3 rs4612666 polymorphism was associated with protection against EPTB. Increased levels of IL-6 or IL-33 and IL-18 or IL-33 were found in TB individuals both without and with HIV carrying the MFA of some selected NLRP3 polymorphisms. In addition, the G/A genotype in the IL-1β rs1143634 polymorphism was associated with TB risk among PLWH. Increased levels of IL-33 were found in TB individuals without and with HIV who were carrying the IL-1β rs1143634 MFA.

The NLPR3 rs4612666 polymorphism has already been associated with rheumatoid arthritis (Cheng et al., 2021) and cardiovascular diseases (Mahendra et al., 2021). The IL-1β rs1143634 polymorphism is associated with susceptibility to myocardial infarction (Fang et al., 2018), an aggressive phenotype of breast cancer (Wang and Yuan, 2022), and is a predictive factor for a severe course of chronic periodontitis (Brodzikowska et al., 2019). To the best of our knowledge, this is the first study to report the association of these polymorphisms with the studied TB and TB-HIV outcomes. More studies are needed to confirm these findings.

It is evident that studying only one gene polymorphism is insufficient to explain the complexity of TB-HIV inflammatory outcomes. However, descriptions of genetic associations, even if at the Single Nucleotide Polymorphism (SNP) level, help understand the complex mechanisms that are involved in infectious diseases. It must be considered that other components of inflammasomes may regulate inflammation, in addition to other host genetic factors that are linked to TB-HIV immunopathogenesis. HLA and KIR alleles associations were previously described by our group (de Sá et al., 2020) and others, that should be considered in the search for genetic biomarkers of inflammatory diseases, including TB-HIV/IRIS. The importance of the selected inflammasome genes justifies the research conducted in the present study and the results obtained, which were generated using a suitable statistical approach, to adequately demonstrate the relationships among inflammasome-mediated innate immunity Single Nucleotide Polymorphism (SNPs) and TB-HIV/IRIS, as well as the occurrence of TB and its clinical presentations.

Several studies have related potential biomarkers to cytokine production as predictors of TB-HIV/IRIS onset. Tan et al. (2015) showed that individuals with TB-IRIS have higher levels of plasma IL-18 both in the pre-cART phase and during TB-HIV/IRIS (Tan et al., 2015). Similarly, Conesa-Botella et al. (2012) reported that in individuals without corticosteroid therapy, the levels of tumor necrosis factor (TNF), interferon-gamma (IFN-γ), and plasma levels of IL-6 and IL-18 were significantly higher in TB-HIV individuals with TB-IRIS than in those without IRIS at week two after starting cART. In contrast only the IFN-γ levels were higher in IRIS individuals at baseline (Conesa-Botella et al., 2012). In the present study, possibly due to the low number of subjects with TB-HIV/IRIS, no increase in the IL-1β and IL-18 cytokines, the typical inflammasome stimulation products, as well as IL-6 was detected for this group, but a trend toward increased IL-33 plasma levels was observed.

IL-6 is a known downstream target of IL-1β that is consistently higher in serum samples from individuals with NLRP3 inflammasome-mediated conditions (Brydges et al., [[NoYear]]; Tanaka et al., 2014). IL-6 is a proinflammatory cytokine with a pleiotropic effect on inflammation, immune response, and hematopoiesis (Tanaka et al., 2014). High levels of IL-6 have been described as a potential biomarker for TB and are associated with higher plasma viral loads and faster progression to AIDS in several studies (Boulware et al., 2011; Singh and Goyal, 2013; Joshi et al., 2015). In this study, IL-6 levels were higher in individuals with TB than those without TB and among PLWH with TB than those without TB.

Regarding IL-33, several studies report that this cytokine acts as an “alarm” that can be released upon tissue damage, stress, or infection, which acts as a danger signal for the immune system (Andreone et al., 2020; Cayrol and Girard, 2018; Neumann et al., 2018). In this study, the IL-33 levels were higher in individuals with TB than in those without TB and in PLWH with TB than those without TB. The role of this cytokine in HIV-1 infection and TB has already been described (Xuan et al., 2014; Wu et al., 2018), which shows potential therapeutic effects on established MTB infections, which might represent a novel therapy for PTB (Piñeros et al., 2017). Zhao et al., 2021 showed that the plasma IL-33 levels were significantly higher in individuals with PTB than in healthy individuals (Zhao et al., 2021).

As mentioned earlier, in addition to inflammasome stimulation and cytokine release in TB-HIV coinfection, these processes can lead to extensive inflammation with cell damage and, consequently, overproduction of IL-33, which increases in those who progress to TB-HIV/IRIS, as suggested by our study. Therefore, additional studies are needed to investigate the roles of CARD8 and AIM2 gene variations in the modulation of inflammasome and cytokine secretion, mainly IL-33, in the context of TB-HIV/IRIS.

In conclusion, our study contributes to the generation of knowledge on the role of inflammasome Single Nucleotide Polymorphism (SNPs) and inflammatory cytokines in TB-HIV outcomes and the evolution toward TB-HIV/IRIS. Nevertheless, it is relevant to note that some limitations of the current study should be considered, mainly concerning the limited sample size and low frequency of HIV/TB-IRIS cases. Therefore, additional studies with larger populations are needed to understand better the importance and roles of inflammasome Single Nucleotide Polymorphism (SNPs) and inflammatory cytokines in TB-HIV/IRIS.

## Data availability statement

The raw data supporting the conclusions of this article will be made available by the authors, without undue reservation.

## Ethics statement

The studies involving human participants were reviewed and approved by IOC/FIOCRUZ (CAAE 51959215.5.0000.5248), INI/FIOCRUZ (CAAE 51959215.5.3002.5262), and HGNI (CAAE 51959215.5.3001.5254) Ethical Boards. The individuals/participants provided their written informed consent to participate in this study. The patients/participants provided their written informed consent to participate in this study.

## Author contributions

NSá: conceptualization, methodology, validation, investigation, writing - original draft and visualization. NSo and MN-G: methodology, validation, and investigation. MR-A: software, formal analysis and resources, and writing - original draft. TS, JP, VR, CG-G, LdOP, and DS-A: resources and writing - review & editing. MM and ST: conceptualization, methodology, writing - original draft, and supervision. All authors read and approved the manuscript.

## Funding

This work was supported by the Conselho Nacional de Desenvolvimento Científico e Tecnológico – CNPq (Grants numbers 404573/2012-6; 311345/2014-0; 435002/2018-0; 314064/2018-4), Fundação Carlos Chagas Filho de Amparo à Pesquisa do Estado do Rio de Janeiro- FAPERJ (Grant number E-26/010.001673/2019), and the France Recherche Nord & Sud Sida-HIV Hépatites - ANRS (Grant number ANRS12274). NBRS is recipient of INOVA FIOCRUZ/ Fundação Oswaldo Cruz postdoctoral fellowship. MGM is recipient of CNPQ (314064/2018-4) and FAPERJ (E-26/201.177/2021) research fellowships.

## Acknowledgments

The authors are thankful to all individuals who agreed to participate in this study as volunteers and permitted the analysis of their biological material. We are in debt to Iury Amâncio Paiva and Jéssica Badolato Corrêa da Silva for technical support.

## Conflict of interest

The authors declare that the research was conducted in the absence of any commercial or financial relationships that could be construed as a potential conflict of interest.

## Publisher’s note

All claims expressed in this article are solely those of the authors and do not necessarily represent those of their affiliated organizations, or those of the publisher, the editors and the reviewers. Any product that may be evaluated in this article, or claim that may be made by its manufacturer, is not guaranteed or endorsed by the publisher.
