# Supplementary material for: Inflammasome genetic variants are associated with tuberculosis, HIV-1 infection, and TB/HIV-immune reconstitution inflammatory syndrome outcomes
Source: Front Cell Infect Microbiol. 2022 Sep 20;12:962059. doi: 10.3389/fcimb.2022.962059 (PMC9531132; doi:10.3389/fcimb.2022.962059)
Supplement: Supplementary file 1 [file Table_1.docx]

**Supplementary data**

**Table S1**: Characteristics of inflammasome SNPs included in the study.

| Gene | SNP ID | Chromosome | Position | Ancestral allele | Variation allele | *P*-value  HWE^a^ | | | |
| --- | --- | --- | --- | --- | --- | --- | --- | --- | --- |
|  |  |  |  |  |  | **With TB *vs*.**  **Without TB** | **PTB *vs*. EPTB** | **PLWH with TB *vs*.**  **Without TB** | **With IRIS *vs*.**  **Without IRIS** |
| CARD8 | rs2043211 | 19 | 48234449 | A | T | 1 | 0.974 | 0.580 | 0.547 |
| CARD8 | rs6509365 | 19 | 48240212 | A | G | 0.980 | 1 | 1 | 0.789 |
| AIM2 | rs2276405 | 1 | 159073406 | C | T | 1 | 1 | 1 | 1 |
| IFI16 | rs1101996 | 1 | 159028236 | A | C | 0.698 | 0.690 | 0.580 | 0.539 |
| CASP1 | rs572687 | 11 | 105032992 | A | G | 0.980 | 0.690 | 0.580 | 0.648 |
| IL-1β | rs1143634 | 2 | 112832813 | G | A | 0.560 | 0.974 | 0.580 | 0.963 |
| NLRP3 | rs3806268 | 1 | 247424175 | A | G | 0.698 | 1 | 0.696 | 0.789 |
| NLRP3 | rs35829419 | 1 | 247425556 | A | C | 1 | 1 | 1 | 1 |
| NLRP3 | rs4612666 | 1 | 247435768 | C | T | 0.980 | 1 | 0.696 | 1 |
| NLRP3 | rs15390193 | 1 | 247436999 | A | C | 0.980 | 1 | 0.901 | 0.963 |
| NLRP3 | rs10754558 | 1 | 247448734 | C | G | 0.560 | 1 | 0.896 | 0.185 |

SNP: single nucleotide polymorphism; CARD8: Caspase Recruitment Domain-Containing Protein 8; AIM2: Absent In Melanoma 2; IFI16: Interferon Gamma Inducible Protein 16; CASP1: Caspase 1; IL-1β: Interleukin 1 Beta; NLRP3: NLR family Pyrin Domain Containing 3. ^a^P-value of the Hardy- Weinberg equilibrium.

**Table S2**: Sociodemographic, clinical, and laboratory data of TB clinical presentations of the individuals included in this study.

| Features | Site of TB | | aOR^a^ (CI95%) | *P*-value^b^ |
| --- | --- | --- | --- | --- |
|  | **PTB**  **N=63** | **EPTB**  **N=49** |  |  |
| Gender; n (%) |  |  |  |  |
| Male | 45 (71.43%) | 41 (83.67%) | Reference | Reference |
| Female | 18 (28.57%) | 8 (16.33%) | 0.59 (0.22-1.58) | 0.294 |
| SkinColor^c^; n (%) |  |  |  |  |
| Brown | 24 (38.1%) | 17 (34.69%) | Reference | Reference |
| Black | 16 (25.4%) | 9 (18.37%) | 0.67 (0.22-2.07) | 0.504 |
| White | 23 (36.51%) | 23 (46.94%) | 1.72 (0.68-4.32) | 0.504 |
| Education^d^; n (%) |  |  |  |  |
| Bachelor | 4 (6.35%) | 2 (4.08%) | 1.02 (0.15-6.93) | 1 |
| Upper-secondary | 17 (26.98%) | 10 (20.41%) | 0.93 (0.32-2.73) | 1 |
| Lower-secondary | 27 (42.86%) | 19 (38.78%) | Reference | Reference |
| Primary | 10 (15.87%) | 16 (32.65%) | 3.08 (1.05-9) | 0.161 |
| Unknown | 5 (7.94%) | 2 (4.08%) | 1.15 (0.16-8.09) | 1 |
| HIV status; n (%) |  |  |  |  |
| Yes | 46 (73.02%) | 42 (85.71%) | Reference | Reference |
| No | 17 (26.98%) | 7 (14.29%) | 0.57 (0.21-1.6) | 0.289 |
| CD4 count (cells/µL); n (%) |  |  |  |  |
| ≤ 200 cells/µL | 38 (61.29%) | 29 (63.04%) | Reference | Reference |
| > 200 cells/µL | 24 (38.71%) | 17 (36.96%) | 1.44 (0.45-4.58) | 0.540 |
| CD8 count (cells/µL); n (%) |  |  |  |  |
| ≤ 500 cells/µL | 30 (50.85%) | 29 (65.91%) | Reference | Reference |
| > 500 cells/µL | 29 (49.15%) | 15 (34.09%) | 0.56 (0.24-1.29) | 0.174 |
| CD4/CD8 ratio; n (%) |  |  |  |  |
| ≤ 1 | 47 (79.66%) | 38 (86.36%) | Reference | Reference |
| > 1 | 12 (20.34%) | 6 (13.64%) | 2.87 (0.26-31.78) | 0.390 |

^a^Odds ratios were adjusted by skin color, education, site of tuberculosis, HIV transmission route, and CD8 count. ^b^P-values were calculated using the unconditional logistic regression model. Associations were considered significant with a value of * P < 0.05. ^c^Skin color categorization followed the classificatory system employed by the Brazilian Institute of Geography and Statistics (IBGE) (40). ^d^Classification, according to the International Standard Classification of Education (ISCED) maintained by the United Nations Educational, Scientific and Cultural Organization (UNESCO). N: number of individuals in each group, TB: tuberculosis, %: Frequencies, aOR: adjusted odds ratio; 95% CI: 95% confidence interval.

**Table S3**: Sociodemographic, clinical, and laboratory data of HIV-1-positive individuals included in the study categorized according to the presence or absence of TB.

| Features | PLWH | | aOR (CI95%) | *P*-value^a^ |
| --- | --- | --- | --- | --- |
|  | **With TB**  **N=88** | **Without TB**  **N=20** |  |  |
| Gender; n (%) |  |  |  |  |
| Male | 70 (79.55%) | 13 (65%) | Reference | Reference |
| Female | 18 (20.45%) | 7 (35%) | 0.41 (0.13-1.3) | 0.130 |
| SkinColor^b^; n (%) |  |  |  |  |
| Brown | 32 (36.36%) | 12 (60%) | Reference | Reference |
| Black | 21 (23.86%) | 3 (15%) | 3.52 (0.78-15.79) | 0.201 |
| White | 35 (39.77%) | 5 (25%) | 2.77 (0.82-9.31) | 0.201 |
| Education^c^; n (%) |  |  |  |  |
| Bachelor | 3 (3.41%) | 1 (5%) | 0.66 (0.05-9.56) | 1 |
| Upper-secondary | 19 (21.59%) | 7 (35%) | 0.72 (0.22-2.32) | 1 |
| Lower-secondary | 38 (43.18%) | 12 (60%) | Reference | Reference |
| Primary | 23 (26.14%) | 0 (0%) | NC | NC |
| Unknown | 5 (5.68%) | 0 (0%) | NC | NC |
| HIV transmission route; n (%) |  |  |  |  |
| Heterosexual | 66 (75%) | 16 (80%) | Reference | Reference |
| MSM | 20 (22.73%) | 4 (20%) | 0.86 (0.22-3.34) | 1 |
| Unknown | 2 (2.27%) | 0 (0%) | NC | NC |
| Log CV | 5.34 (IQR=0.96) | 5.22 (IQR=0.51) | 1.11 (0.47-2.61) | 0.817 |
| CD4 count (cells/µL); n (%) |  |  |  |  |
| ≤ 50 cells/µL | 28 (33.33%) | 11 (55%) | Reference | Reference |
| <50 cells/µL | 56 (66.67%) | 9 (45%) | 1.48 (0.47-4.69) | 0.504 |
| CD8 count (cells/µL); n (%) |  |  |  |  |
| ≤ 500 cells/µL | 29 (36.71%) | 12 (60%) | Reference | Reference |
| > 500 cells/µL | 50 (63.29%) | 8 (40%) | 2.62 (0.8-8.53) | 0.110 |
| CD4/CD8 ratio; n (%) |  |  |  |  |
| ≤ 0.15 | 46 (58.23%) | 12 (60%) | Reference | Reference |
| >0.15 | 33 (41.77%) | 8 (40%) | 0.66 (0.15-3) | 0.590 |

^a^Odds ratios were adjusted by skin color, education, site of tuberculosis, HIV transmission route, and CD8 count. ^b^P-values were calculated using the unconditional logistic regression model. Associations were considered significant with a value of * P < 0.05. ^c^Skin color categorization followed the classificatory system employed by the Brazilian Institute of Geography and Statistics (IBGE) (40). ^d^Classification, according to the International Standard Classification of Education (ISCED) maintained by the United Nations Educational, Scientific and Cultural Organization (UNESCO). N: number of individuals in each group, TB: tuberculosis, PLWH: People Living with HIV, %: Frequencies, aOR: adjusted odds ratio; 95% CI: 95% confidence interval.

**Table S4**: Sociodemographic, clinical, and laboratory data of TB-HIV individuals.

| **Features** | **Without IRIS**  **N=77** | **With IRIS**  **N=11** | **aOR^a^ (CI95%)** | ***P*-value^b^** |
| --- | --- | --- | --- | --- |
| **Gender; n (%)** |  |  |  |  |
| **Male** | 59 (76.62%) | 11 (100%) | Reference | |
| **Female** | 18 (23.38%) | 0 (0%) | NC | |
| **Skin Color^c^; n (%)** |  |  |  |  |
| **Brown** | 30 (38.96%) | 2 (18.18%) | Reference | |
| **Black** | 18 (23.38%) | 3 (27.27%) | 6.34 (0.38-106.84) | 0.380 |
| **White** | 29 (37.66%) | 6 (54.55%) | 5.42 (0.43-67.71) | 0.380 |
| **Education^d^; n (%)** |  |  |  |  |
| **Bachelor** | 2 (2.6%) | 1 (9.09%) | 7.58 (0.15-392.61) | 0.944 |
| **Upper-secondary** | 17 (22.08%) | 2 (18.18%) | 0.34 (0.03-4.17) | 0.944 |
| **Lower-secondary** | 31 (40.26%) | 7 (63.64%) | Reference | |
| **Primary** | 22 (28.57%) | 1 (9.09%) | 0.12 (0.01-2.42) | 0.666 |
| **Unknown** | 5 (6.49%) | 0 (0%) | NC | |
| **Site of Tuberculosis; n (%)** |  |  |  |  |
| **Pulmonary** | 43 (55.84%) | 3 (27.27%) | Reference | |
| **Extrapulmonary** | 34 (44.16%) | 8 (72.73%) | 6.6 (1.11-39.33) | **0.038** |
| **HIV transmission route; n (%)** |  |  |  |  |
| **Heterosexual/Oth** | 61 (79.22%) | 5 (45.45%) | Reference | |
| **MSM** | 15 (19.48%) | 5 (45.45%) | 3.35 (0.63-17.92) | 0.315 |
| **Unknown** | 1 (1.3%) | 1 (9.09%) | NC | |
| **CD4 count (cell/µL) (IQR)** |  |  |  |  |
| **(≤50); n (%)** | 23 (31.08%) | 5 (50%) | 0.75 (0.12-4.85) | 0.766 |
| **(>50); n (%)** | 51 (68.92%) | 5 (50%) | Reference | |
| **CD8 count (IQR)** |  |  |  |  |
| **(≤500); n (%)** | 22 (31.88%) | 7 (70%) | 12.32 (1.82-83.55) | **0.010** |
| **(>500); n (%)** | 47 (68.12%) | 3 (30%) | Reference | |
| **CD4/CD8 ratio (IQR)** |  |  |  |  |
| **(≤0.15); n (%)** | 39 (56.52%) | 7 (70%) | Reference | |
| **(>0.15); n (%)** | 30 (43.48%) | 3 (30%) | 0.27 (0.03-2.32) | 0.235 |
| **Viral load (copies/mL) (IQR)** |  |  |  |  |
| **(<20000); n (%)** | 35 (50%) | 4 (40%) | Reference | |
| **(>20000); n (%)** | 35 (50%) | 6 (60%) | 0.1 (0-2.39) | 0.157 |

^a^Odds ratio were adjusted by skin color, education, site of tuberculosis, HIV transmission route, and CD8 count. ^b^P-values were calculated using Fisher’s exact test. Associations were considered significant with a value of * P < 0.05. ^c^Skin color categorization followed the classificatory system employed by the Brazilian Institute of Geography and Statistics (IBGE) (40). ^d^Classification, according to the International Standard Classification of Education (ISCED) maintained by the United Nations Educational, Scientific and Cultural Organization (UNESCO). N: number of individuals in each group, %: Frequencies, aOR: adjusted odds ratio; 95% CI: 95% confidence interval.

**Table S5**: Cytokines plasma levels according to the evaluated SNPs between TB-HIV individuals.

| Cytokines | Gene SNP (rs) | Carrier | Mean (CI95%) | | P-value |
| --- | --- | --- | --- | --- | --- |
|  |  |  | **With IRIS** | **Withou IRIS** |  |
| IL-1β | CARD8 rs2043211 | A | -0.018 (-0.574 - 0.538) | 0.19 (-0.022 - 0.403) | 0.873 |
|  | CARD8 rs6509365 | A | 0.053 (-0.347 - 0.454) | 0.182 (-0.031 - 0.395) | 0.906 |
|  | AIM2 rs2276405 | C | 0.12 (-0.45 - 0.69) | 0.165 (-0.411 - 0.741) | 0.999 |
|  | CASP-1 rs572687 | G | 0.046 (-0.312 - 0.403) | 0.081 (-0.165 - 0.328) | 0.997 |
|  | IFI16 rs11019966 | C | -0.023 (-0.35 - 0.304) | 0.135 (-0.122 - 0.392) | 0.890 |
|  | IL-1β rs1143634 | G | 0.037 (-0.228 - 0.302) | 0.14 (-0.115 - 0.395) | 0.933 |
|  | NLRP3 rs3806268 | G | 0.046 (-0.27 - 0.363) | 0.17 (-0.076 - 0.415) | 0.780 |
|  | NLRP3 rs4612666 | C | -0.006 (-0.254 - 0.242) | 0.084 (-0.143 - 0.311) | 0.929 |
|  | NLRP3 rs1539019 | C | 0.022 (-0.215 - 0.258) | 0.15 (-0.074 - 0.374) | 0.762 |
|  | NLRP3 rs10754558 | C | -0.054 (-0.388 - 0.28) | 0.113 (-0.114 - 0.34) | 0.622 |
| IL-6 | CARD8 rs2043211 | A | 1.855 (0.963 - 2.747) | 1.003 (0.661 - 1.345) | 0.228 |
|  | CARD8 rs6509365 | A | 1.363 (0.693 - 2.032) | 0.951 (0.599 - 1.303) | 0.570 |
|  | AIM2 rs2276405 | C | 0.818 (-0.132 - 1.769) | 0.734 (-0.23 - 1.698) | 0.999 |
|  | CASP-1 rs572687 | G | 0.947 (0.343 - 1.551) | 0.968 (0.552 - 1.385) | 1,000 |
|  | IFI16 rs11019966 | C | 1.464 (0.932 - 1.996) | 0.812 (0.395 - 1.23) | 0.267 |
|  | IL-1β rs1143634 | G | 1.213 (0.767 - 1.658) | 0.951 (0.535 - 1.367) | 0.794 |
|  | NLRP3 rs3806268 | G | 0.724 (0.219 - 1.229) | 0.763 (0.379 - 1.147) | 0.998 |
|  | NLRP3 rs4612666 | C | 1.249 (0.824 - 1.673) | 0.955 (0.57 - 1.34) | 0.651 |
|  | NLRP3 rs1539019 | C | 0.969 (0.581 - 1.356) | 1.076 (0.711 - 1.442) | 0.958 |
|  | NLRP3 rs10754558 | C | 1.475 (0.956 - 1.995) | 1.205 (0.85 - 1.561) | 0.612 |
| IL-18 | CARD8 rs2043211 | A | 1.809 (0.862 - 2.756) | 1.66 (1.296 - 2.024) | 0.989 |
|  | CARD8 rs6509365 | A | 1.402 (0.725 - 2.078) | 1.603 (1.246 - 1.961) | 0.923 |
|  | AIM2 rs2276405 | C | 0.982 (0.075 - 1.89) | 1.383 (0.462 - 2.304) | 0.915 |
|  | CASP-1 rs572687 | G | 1.677 (1.088 - 2.266) | 1.74 (1.333 - 2.146) | 0.996 |
|  | IFI16 rs11019966 | C | 1.487 (0.964 - 2.011) | 1.842 (1.431 - 2.252) | 0.742 |
|  | IL-1β rs1143634 | G | 1.929 (1.498 - 2.36) | 1.654 (1.252 - 2.056) | 0.749 |
|  | NLRP3 rs3806268 | G | 1.603 (1.085 - 2.121) | 1.685 (1.291 - 2.079) | 0.980 |
|  | NLRP3 rs4612666 | C | 1.81 (1.397 - 2.223) | 1.64 (1.265 - 2.014) | 0.898 |
|  | NLRP3 rs1539019 | C | 1.519 (1.145 - 1.893) | 1.753 (1.4 - 2.105) | 0.672 |
|  | NLRP3 rs10754558 | C | 1.74 (1.186 - 2.294) | 1.714 (1.331 - 2.096) | 0.999 |
| IL-33 | CARD8 rs2043211 | A | 2.424 (1.05 - 3.799) | 1.187 (0.66 - 1.713) | 0.276 |
|  | CARD8 rs6509365 | A | 1.938 (0.938 - 2.938) | 1.205 (0.679 - 1.731) | 0.418 |
|  | AIM2 rs2276405 | C | 1.407 (-0.014 - 2.828) | 1.664 (0.222 - 3.106) | 0.993 |
|  | CASP-1 rs572687 | G | 1.916 (1.011 - 2.82) | 1.343 (0.719 - 1.967) | 0.547 |
|  | IFI16 rs11019966 | C | 2.099 (1.295 - 2.903) | 1.216 (0.585 - 1.847) | 0.361 |
|  | IL-1β rs1143634 | G | 1.75 (1.084 - 2.416) | 1.387 (0.765 - 2.009) | 0.829 |
|  | NLRP3 rs3806268 | G | 1.371 (0.588 - 2.155) | 1.116 (0.52 - 1.711) | 0.857 |
|  | NLRP3 rs4612666 | C | 1.827 (1.191 - 2.463) | 1.221 (0.645 - 1.798) | 0.384 |
|  | NLRP3 rs1539019 | C | 1.794 (1.201 - 2.388) | 1.366 (0.806 - 1.925) | 0.562 |
|  | NLRP3 rs10754558 | C | 1.936 (1.155 - 2.717) | 1.518 (0.984 - 2.053) | 0.589 |

P-values were calculated using the unconditional logistic regression model. Associations were considered significant with a value of * P < 0.05. aOR: adjusted odds ratio; 95% CI: 95% confidence interval. The NLRP3 rs35829419 had insufficient observations and/or no way to calculate the standard error (all observations from one or more groups were equal to the lower detection limit of the assay) for the analyses between the groups with *vs*. without TB, with TB *vs*. without TB among HIV and PTB *vs*. EPTB.
